# Supplementary material for: Empirical methods for controlling false positives and estimating confidence in ChIP-Seq peaks
Source: BMC Bioinformatics. 2008 Dec 5;9:523. doi: 10.1186/1471-2105-9-523 (PMC2628906; doi:10.1186/1471-2105-9-523)
Supplement: Additional File 1 — A variety of html documents from the USeq web site detailing the available applications, their best usage, output file type descriptions, command line menus, etc. [file 1471-2105-9-523-S1.zip › USeqUserGuides/JavaDoc.zip!/JavaDoc/trans/roc/ParseBarsForParticularRegions.html]

ParseBarsForParticularRegions


|  |  |  |
| --- | --- | --- |
| **Overview** \| **Package** \| **Class** \| **Use** \| **Tree** \| **Deprecated** \| **Index** \| **Help** | |  |
| **PREV CLASS**   **NEXT CLASS** | **FRAMES**    **NO FRAMES**     **All Classes** |  |
| SUMMARY: NESTED \| FIELD \| CONSTR \| METHOD | DETAIL: FIELD \| CONSTR \| METHOD |  |


---


## trans.roc Class ParseBarsForParticularRegions

```
java.lang.Object
  trans.roc.ParseBarsForParticularRegions
```

---

``` public class ParseBarsForParticularRegions extends java.lang.Object ```

Returns oligo values that overlap particular regions.

---

**Constructor Summary**

`ParseBarsForParticularRegions(java.io.File barDirectory,
int bpPositionOffSetBar,
int bpPositionOffSetRegion)`

`ParseBarsForParticularRegions(java.lang.String[] args)`


| **Method Summary** | |
| --- | --- |
| `double` | `calculateFractionGCContent(int start, int stop)`             Calculates the gc content. |
| `void` | `calculateMedians(UCSCGeneLine[] lines)`             Calculates the median intensity for the oligos that lie under a gene. |
| `java.util.ArrayList` | `fetchRegionGrs(java.lang.String chr, int start, int stop)`             Fetches the Grs under the region, Uses the bpPositionOffSets so set appropriately! |
| `static int` | `inRegion(Sgr sgr, Positive[] pos)`             Checks to see if an sgr object is in one of the positives, if not returns -1, otherwise returns index number. |
| `void` | `loadChromosomeBarFile()` |
| `void` | `loadRegion(Positive region)`             Loads a Positive with Gr objects. |
| `static void` | `main(java.lang.String[] args)` |
| `void` | `makeBarFileHashMap()` |
| `void` | `makeRandom(Positive region, double numberScores)`             Takes a Positive and finds 1000 chrom, length, gc, and number scores matched random regions. |
| `static void` | `printDocs()` |
| `void` | `processArgs(java.lang.String[] args)`             This method will process each argument and assign new varibles |

**Methods inherited from class java.lang.Object**

`equals, getClass, hashCode, notify, notifyAll, toString, wait, wait, wait`

**Constructor Detail**

### ParseBarsForParticularRegions

```
public ParseBarsForParticularRegions(java.io.File barDirectory,
                                     int bpPositionOffSetBar,
                                     int bpPositionOffSetRegion)
```

---


### ParseBarsForParticularRegions

```
public ParseBarsForParticularRegions(java.lang.String[] args)
```


**Method Detail**

### fetchRegionGrs

```
public java.util.ArrayList fetchRegionGrs(java.lang.String chr,
                                          int start,
                                          int stop)
```

:   Fetches the Grs under the region,
    Uses the bpPositionOffSets so set appropriately!

---


### calculateMedians

```
public void calculateMedians(UCSCGeneLine[] lines)
```

:   Calculates the median intensity for the oligos that lie under a gene.

---


### makeRandom

```
public void makeRandom(Positive region,
                       double numberScores)
```

:   Takes a Positive and finds 1000 chrom, length, gc, and number scores matched random regions.
    Calculates each mean and sets in the Positive.

---


### calculateFractionGCContent

```
public double calculateFractionGCContent(int start,
                                         int stop)
```

:   Calculates the gc content.

---


### loadChromosomeBarFile

```
public void loadChromosomeBarFile()
```

---


### inRegion

```
public static int inRegion(Sgr sgr,
                           Positive[] pos)
```

:   Checks to see if an sgr object is in one of the positives, if not returns -1,
    otherwise returns index number.

---


### loadRegion

```
public void loadRegion(Positive region)
```

:   Loads a Positive with Gr objects.

---


### main

```
public static void main(java.lang.String[] args)
```

---


### processArgs

```
public void processArgs(java.lang.String[] args)
```

:   This method will process each argument and assign new varibles

---


### makeBarFileHashMap

```
public void makeBarFileHashMap()
```

---


### printDocs

```
public static void printDocs()
```


---


|  |  |  |
| --- | --- | --- |
| **Overview** \| **Package** \| **Class** \| **Use** \| **Tree** \| **Deprecated** \| **Index** \| **Help** | |  |
| **PREV CLASS**   **NEXT CLASS** | **FRAMES**    **NO FRAMES**     **All Classes** |  |
| SUMMARY: NESTED \| FIELD \| CONSTR \| METHOD | DETAIL: FIELD \| CONSTR \| METHOD |  |


---
